# Fostering life hope in urban green spaces through brief online mindfulness: findings from four studies with park visitors

**DOI:** 10.3389/fpsyg.2025.1642533

**Published:** 2025-07-23

**Authors:** Mengke Luo, Weiyi Zhang

**Affiliations:** ^1^Macau University of Science and Technology, Avenida WaiLong,Taipa, Macao SAR, China; ^2^Huainan Normal University, West Dongshan Road, Tianjiaan District, Huainan, China

**Keywords:** urban green spaces, mindfulness training, life hope, flow, sense of meaning in life

## Abstract

Contrary to the prevailing view that technology detracts from nature, this research demonstrates that digitally-guided mindfulness can act as a powerful amplifier of the psychological benefits of Urban Green Spaces (UGS). Across four interconnected experiments, we reveal how and for whom this synergy works to enhance life hope. Building on Attention Restoration, Broaden-and-Build, and Hope theories, our investigation shows that an online mindfulness intervention in a park setting significantly boosts life hope more than a self-guided offline practice (Experiment 1). This effect is mediated by the induction of flow, a state of deep absorption in the present moment (Experiment 2). Furthermore, our model reveals crucial boundary conditions: this positive, flow-driven pathway is significantly stronger for individuals experiencing higher levels of spirituality (Experiment 3) and possessing a greater sense of meaning in life (Experiment 4). Collectively, these findings offer a novel, evidence-based framework showing that personalized, digitally-delivered interventions can transform UGS into more effective therapeutic landscapes. This provides actionable insights for urban planners and digital health developers to create scalable, accessible, and highly effective nature-based solutions for promoting public mental wellbeing. Future research should address limitations by incorporating objective measures and examining long-term effects.

## 1 Introduction

Urban green spaces (UGS) are increasingly celebrated not merely as ecological assets but as crucial sites for psychological restoration and enhancing the quality of urban tourism experiences (Kuznetsova et al., 2024). While the restorative potential of nature engagement within these oases is well-documented (Ruffatto et al., 2022), a subtle yet significant disjuncture emerges: the passive consumption of these environments may not spontaneously or sufficiently translate into the cultivation of deeper, more enduring psychological resources such as life hope, particularly for visitors navigating the pervasive stressors of contemporary urban living. This gap, between the inherent salutogenic promise of UGS and the actualized fostering of profound individual strengths, underscores the necessity of proactive strategies within these green settings to actively nurture such vital psychological assets (Zhou et al., 2024). Against this backdrop, leveraging UGS as platforms for accessible psychological interventions like online mindfulness training becomes paramount, as the capacity of such training to directly address and potentially elevate life hope during the park visit experience itself warrants significant scholarly attention.

The extant literature offers compelling, albeit largely separate, streams of evidence supporting the core components of this inquiry. Research robustly demonstrates the capacity of UGS to mitigate stress and enhance mood (Wrapson et al., 2021), yet the focus has often been on general affective restoration rather than the deliberate cultivation of specific positive psychological constructs like life hope (Zarzycka et al., 2024). Separately, mindfulness interventions have garnered significant empirical support for their efficacy in fostering psychological well-being (Santos et al., 2024), reducing rumination (Atashipour et al., 2023), and even enhancing hope in various populations. However, the application of mindfulness, particularly online modalities, within the specific experiential context of UGS visitation, and its direct impact on visitor life hope (Fu et al., 2025), remains a nascent area of investigation. While some studies explore nature-based mindfulness (e.g., forest bathing), they seldom employ online delivery during an actual park visit nor focus explicitly on life hope as a primary outcome through experimental designs with urban park visitors (Precup, 2025). This confluence of established benefits and unaddressed specificities highlights a critical gap: a lack of empirical understanding regarding whether a targeted, accessible intervention like online mindfulness training can indeed enhance the life hope of individuals while they are engaging with urban green spaces.

To bridge a significant gap in understanding the utility of online mindfulness within Urban Green Spaces (UGS), this research encompasses a series of four interconnected experiments conducted in simulated urban park environments. Experiment 1 initiated this line of inquiry by contrasting the effectiveness of online mindfulness training against its traditional offline counterpart in promoting participants’ life hope, thereby assessing the viability of digital delivery. Subsequently, Experiment 2 delved into the psychological underpinnings, specifically investigating flow as a mediating mechanism in the relationship between mindfulness training modality (online vs. offline) and resultant life hope. The research further explored moderating factors influencing this mediated pathway. Experiment 3 examined the moderating role of ‘spirituality’ on the indirect effect of mindfulness training on life hope via flow. Lastly, Experiment 4 expanded on this moderated mediation model by evaluating the distinct and potentially synergistic moderating impacts of both ‘spirituality’ and ‘Meaning in Life’ on the mindfulness-flow-life hope connection. Collectively, these investigations aim to furnish a comprehensive empirical understanding not only of whether online mindfulness training augments life hope in UGS settings, but critically, the process by which this occurs (mediated by flow) and the psychological contexts (moderated by spirituality and meaning in life) under which these advantages are maximized.

The anticipated theoretical contributions of this multi-experiment investigation are substantial and multifaceted, aiming to significantly advance our understanding of how positive psychological states like life hope can be actively cultivated within urban green space tourism contexts (Wang J. et al., 2024; Wang S. M. et al., 2024). Firstly, by empirically validating the efficacy of online mindfulness training in enhancing life hope among UGS visitors (Experiment 1), this research extends existing theories on restorative environments and digital well-being interventions, demonstrating the potential to transcend passive nature exposure by integrating accessible, active psychological tools (Wright et al., 2024). Secondly, the elucidation of Flow as a key mediating mechanism (Experiment 2) between mindfulness training (both online and offline) and life hope provides a crucial process-oriented insight, bridging mindfulness theory with Csikszentmihalyi’s flow theory and positive psychology frameworks on hope development within an experiential tourism setting (Sinéad et al., 2024). Thirdly, and perhaps most significantly, the systematic investigation into the moderating roles of ‘spirituality’ (Experiment 3) and the combined influence of ‘spirituality’ and ‘Meaning in Life’ (Experiment 4) on the mindfulness-flow-life hope pathway articulates a more nuanced and contextually sensitive model (Clemente, 2025). This delineates critical psychological conditions under which the salutogenic effects of mindfulness in UGS are amplified, thereby contributing to a more sophisticated understanding of person-environment-intervention interactions. Collectively, this research aims to pioneer a more holistic theoretical framework that integrates digital mental health strategies with environmental psychology and tourism experience design, specifically for fostering enduring positive psychological capital like life hope (Liu et al., 2025). Practically, these insights can inform the design and targeted deployment of online mindfulness tools by UGS managers and tourism operators to proactively enhance visitor well-being.

## 2 Theoretical background and research hypotheses

### 2.1 Theoretical background

#### 2.1.1 Attention restoration theory

According to Attention Restoration Theory (ART) from Stephen and Rachel Kaplan, exposure to specific environments, particularly nature, can promote restoration from cognitive fatigue (Tsai, 2025). The core tenet of ART is that directed attention—the ability to consciously focus and sustain mental effort—is a finite resource that becomes depleted after prolonged cognitive tasks or coping with stressors, leading to reduced efficiency and mental fatigue (Zhu et al., 2024). ART posits that exposure to environments capable of eliciting involuntary or inherent attention allows the fatigued directed attention mechanisms to rest and replenish. Such restorative environments typically possess four key characteristics: Being Away, a sense of psychological or physical distance from daily routines or stressors; Extent, the quality of an environment being rich and coherent enough to engage the mind and support exploration (Jeong, 2024); Fascination, elements within the environment that effortlessly draw attention, particularly “soft fascination” (e.g., subtle changes in natural landscapes) which allows for reflective thought (Tondo, 2023); and Compatibility, a high degree of fit between the environmental features and an individual’s goals, needs, and inclinations, allowing them to feel comfortable and at ease.

Within the research context of “Fostering Life Hope in Urban Green Spaces: An Experimental experiment on the Effects of Online Mindfulness Training for Park Visitors,” Attention Restoration Theory provides a robust theoretical foundation for understanding the role of urban green spaces (UGS) in promoting visitor psychological well-being and their potential synergy with online mindfulness interventions (Zhao, 2024). Firstly, UGS, by their natural attributes, inherently often embody the four restorative characteristics described by ART: park environments allow visitors to achieve “being away” from urban bustle; their diverse landscape elements and spatial layouts offer “extent”; the dynamic and static beauty of nature (e.g., swaying trees, birdsong) constitutes powerful “soft fascination”; and the functions of parks (e.g., rest, walking) are highly “compatible” with visitors’ leisure needs (An et al., 2024). Therefore, according to ART, UGS themselves are ideal settings for promoting visitors’ attention restoration, alleviating cognitive load, and reducing stress (Oluwajana et al., 2023). This state of cognitive restoration provided by UGS creates a more conducive psychological condition for visitors to subsequently engage in online mindfulness training, which requires focused attention, as an individual with partially restored attention may find it easier to enter and maintain a mindful state (Bath and Collier, 2021). Furthermore, the “soft fascination” of UGS can mutually enhance the non-judgmental awareness of present-moment experiences central to mindfulness practice, where natural stimuli in the environment can become positive objects of mindful awareness rather than distractions, thereby potentially deepening the mindfulness training experience and laying the groundwork for the subsequent cultivation of life hope, a more profound positive psychological resource, through mindfulness (Razmus, 2025).

#### 2.1.2 Broaden-and-build theory of positive emotions

Barbara Fredrickson’s Broaden-and-Build Theory of Positive Emotions (Roth and Laireiter, 2021) proposes a distinct function for positive emotions in contrast to negative ones. Unlike negative emotions, which typically restrict an individual’s immediate cognitive and behavioral options to facilitate urgent survival responses (like fight or flight), positive emotions such as joy, interest, and gratitude are hypothesized to expand these momentary repertoires. This expansion increases the range of accessible thoughts and actions. Subsequently, such broadened perspectives can foster the development of lasting personal assets, encompassing physical (e.g., enhanced health), social (e.g., more robust relationships), intellectual (e.g., augmented knowledge, mental agility), and psychological domains (Aydn Kl and Ercokun, 2024). Thus, the theory suggests a dynamic process where fleeting positive emotional experiences accumulate and compound to build lasting psychological capital. In the context of this research, online mindfulness training conducted within the restorative setting of an urban green space is hypothesized to elicit positive emotions (Leong et al., 2024). These emotions, according to the Broaden-and-Build Theory, can expand visitors’ cognitive perspectives and behavioral inclinations (Wang J. et al., 2024; Wang S. M. et al., 2024). This broadening effect, in turn, can facilitate the development of a more hopeful outlook by fostering creative problem-solving for pathways to goals (enhancing Snyder’s pathways thinking) and by building self-efficacy and motivation (enhancing agency thinking), ultimately contributing to an increase in life hope (Fu et al., 2025). Furthermore, experiences of flow and spirituality, which are also positive affective states, can be understood through this lens as potent catalysts for broadening and building.

#### 2.1.3 Hope theory

Hope Theory, as advanced by Snyder and his team (James, 2021), defines hope beyond mere emotion, characterizing it as a goal-driven cognitive framework. This framework underscores an individual’s perceived competence in both discovering pathways to their aspirations and energizing themselves to follow these pathways (Cetin et al., 2024). Within Snyder’s conceptualization, hope comprises two distinct yet interrelated cognitive facets: Pathways thinking and Agency thinking. The former, Pathways thinking, relates to a person’s assessment of their ability to generate viable plans or methods to reach their goals—essentially the “will” to map a course. Agency thinking, conversely, is the impetus component, reflecting one’s perceived ability and determination to use these identified plans to advance towards their goals, symbolizing the “willpower” to persevere (Correll et al., 2021). Those who exhibit strong hope are distinguished by their capacity to devise multiple effective strategies for their goals and to uphold a resilient sense of agency despite obstacles, often by creatively developing alternative approaches. This cognitive framework provides a robust lens through which to understand and measure life hope, the primary outcome variable in the current research, and to explore how interventions like online mindfulness training within urban green spaces might cultivate it (Charness et al., 2024). By enhancing self-awareness, cognitive flexibility, and a present-moment focus, mindfulness training may directly bolster both the ability to envision viable pathways (pathways thinking) and the self-efficacy and motivation to pursue them (agency thinking), thereby fostering an increase in overall life hope among park visitors.

### 2.2 The integrated theoretical framework

To fully leverage the explanatory power of our theoretical foundation, we explicitly articulate the synergistic function of integrating Attention Restoration Theory (ART), the Broaden-and-Build (B&B) Theory, and Hope Theory. We contend that no single theory can adequately explain the full pathway from engaging with a natural environment to cultivating life hope; only through their integration can a comprehensive causal mechanism be understood. Attention Restoration Theory (ART) provides the foundational “Why here?” of our model, explaining why urban green spaces are optimal contexts for psychological restoration by alleviating cognitive fatigue (Tsai, 2025). However, ART’s explanatory power is primarily focused on cognitive restoration and does not, on its own, detail the process by which this restored state translates into specific positive emotions or goal-oriented motivations. It sets the stage but does not describe the full psychological narrative. To bridge this gap, the Broaden-and-Build Theory explains the “How?” of this transformation. It elucidates how the positive emotions elicited by the environment and the mindfulness practice broaden individuals’ thought-action repertoires, thereby building lasting psychological resources (Roth et al., 2024; Santos and Haynos, 2023). Yet, while B&B theory explains the accumulation of general psychological resources, it is less specific about how these broad resources are channeled into the structured, goal-directed cognitions that constitute hope. This is the precise contribution of Hope Theory, which serves as the “What for?” of our model. It provides the necessary specificity by defining the exact psychological resource being cultivated—life hope—through the interplay of pathways and agency thinking (Li et al., 2024). It operationalizes the outcome, explaining how the general resources built by positive emotions are converted into the specific cognitive architecture of hope.

In summary, this integrated framework functions as a cohesive logical chain. ART explains the contextual trigger for mental restoration. B&B theory provides the affective mechanism that transforms cognitive gains into general resources. Hope Theory specifies the motivational outcome, detailing how these resources are structured into life hope. Therefore, the combination of these theories offers a far more comprehensive and nuanced explanation than any single theory could, accounting for the entire process from environmental trigger to specific psychological outcome.

### 2.3 Research hypotheses

#### 2.3.1 Main effect of mindfulness training modality on life hope

Numerous psychological benefits, including enhanced emotional regulation, reduced stress levels, and greater overall life satisfaction, are robustly associated with mindfulness training, a disciplined practice of non-judgmental attention to ongoing experiences (Han and Xu, 2021; Hartzell et al., 2021; Martin et al., 2024; Tudor et al., 2021). The mechanisms for these gains can be partly understood through Broaden-and-Build Theory (Fisher et al., 2022), which posits that mindfulness promotes positive emotions (like calmness and focus); these emotions then broaden an individual’s scope of thought and action, contributing to the accumulation of enduring resources such as life hope. Furthermore, Hope Theory (Park et al., 2025) provides a complementary perspective, proposing that because life hope comprises pathways thinking (discovering routes to goals) and agency thinking (the drive to pursue them), mindfulness may reinforce these cognitive foundations by fostering cognitive adaptability and a stronger sense of self-efficacy.

Urban green spaces (UGS), as environments with restorative qualities (Zhu et al., 2022), can alleviate cognitive fatigue and provide an ideal external setting for mindfulness practice. Online mindfulness training, with its convenience, accessibility, and flexibility, enables immediate mindfulness interventions within UGS, potentially maximizing the synergistic effects of the natural environment and mindfulness practice. In contrast, traditional offline mindfulness training, despite its advantages (Mkinen et al., 2024), might face more organizational and implementation challenges for immediate application in UGS contexts.

Considering that online mindfulness training can be more seamlessly integrated into the immediate experience of urban park visitors, allowing for personalized practice while engaging with nature, we anticipate that the online modality may demonstrate effects comparable to, or even superior to, the traditional offline modality in this specific context. More fundamentally, we first need to verify whether both modalities of mindfulness training can effectively enhance life hope (Schuman-Olivier et al., 2024). Therefore, we propose the following hypotheses:

*H1*: In the context of urban green spaces, the modality of mindfulness training (online vs. offline) will significantly impact park visitors’ life hope. Specifically:

*H1a*: Park visitors participating in online mindfulness training will report significantly higher levels of life hope compared to offline group.

*H1b*: Park visitors participating in offline mindfulness training will report significantly higher levels of life hope compared to offline group.

*H1c*: Park visitors participating in online mindfulness training will report significantly higher levels of life hope compared to those participating in offline mindfulness training.

#### 2.3.2 The mediating role of flow in the relationship between mindfulness training and life Hope

Flow represents a peak mental condition where individuals are completely absorbed and intensely focused on their current task, deriving deep satisfaction and a feeling of command (Kari et al., 2024). Mihaly Csikszentmihalyi characterized flow as a “seamless integration of doing and knowing,” a state commonly involving clear objectives, direct feedback, heightened concentration, diminished self-awareness, an altered perception of time, and inherent gratification from the engagement itself (Merlin and Soubramanian, 2024). Beyond immediate positive feelings and enhanced task execution, flow states are also considered instrumental in promoting enduring personal development and overall well-being (Feng and Zhong, 2021).

Mindfulness training, which emphasizes focused attention and acceptance of present-moment experiences, shares an intrinsic alignment with core characteristics of the flow state, such as concentrated attention and immersion in the current activity (Si et al., 2024). Through mindfulness practice, individuals learn to reduce distractions and engage more deeply with the task or experience at hand, potentially creating favorable conditions for the emergence of flow (Bi et al., 2024). Conducting mindfulness training in urban green spaces (UGS), environments with restorative and “soft fascination” qualities, may more readily disengage visitors’ attention from daily hassles and focus it on the present natural experience and mindfulness guidance, thereby increasing the likelihood of experiencing flow (Johnson et al., 2023).

The experience of flow frequently instills perceptions of enhanced competence, direction, and inherent enjoyment. From the perspective of Broaden-and-Build Theory (Xia and Liuna, 2023), such intensely positive states like flow can expand an individual’s cognitive and behavioral repertoire. This expansion might present as greater self-assurance and an increased propensity for exploration, which directly correspond to the agency and pathways elements within Hope Theory (Colla et al., 2023). Consequently, the positive affect and achievements gained during flow could bolster goal-directed motivation and illuminate routes to goal attainment. On this basis, we propose that flow serves as an intermediary in the connection between mindfulness training (whether delivered online or offline, with Experiment 1 evaluating its immediate impact on life hope) and the level of life hope reported by park visitors.

*H2*: Flow mediates the relationship between mindfulness training and park visitors’ life hope. Specifically:

*H2a*: Mindfulness training (online or offline) will significantly positively predict park visitors’ Flow.

*H2b*: Park visitors’ Flow will significantly positively predict their life hope levels.

*H2c*: Flow will significantly mediate the positive effect of mindfulness training on park visitors’ life hope.

#### 2.3.3 The moderating role of spirituality in the relationships among mindfulness training, flow, and life hope

Spirituality, understood as a multifaceted emotional experience, is typically triggered by stimuli that feel vast, extend beyond one’s usual conceptual frameworks, and prompt cognitive reframing (Liu et al., 2024). These triggers can range from the physically imposing to the conceptually deep or socially significant (Errbii et al., 2024). Experiencing spirituality often leads to a diminished self-focus, increased prosocial inclinations, and a greater appreciation of the present. Urban natural environments (UGS), with their grand vistas, intricate ecological patterns, or the sheer impact of nature’s beauty and power, serve as potent contexts for eliciting spirituality (Shen et al., 2024). Mindfulness, by encouraging receptive attention, may make individuals more sensitive to these awe-inspiring aspects. As a profound positive emotion, spirituality’s capacity to broaden thought-action repertoires and build resources aligns with Broaden-and-Build Theory (Stanley and Schutte, 2023). Consequently, this experiment investigates how spirituality might moderate the effect of mindfulness training on flow, and in turn, the effect of flow on life hope.

Regarding the moderation of spirituality on “Mindfulness Training → Flow”: When individuals engage in mindfulness training in UGS, if they experience higher levels of spirituality (e.g., being deeply moved by the grandeur or intricacy of nature), this profound emotional experience may enhance their immersion and concentration (Lee and Li, 2023). Spirituality can effectively capture attention, reduce mental chatter, and promote a deep connection with the environment, all of which are key elements for entering a state of flow. Therefore, higher levels of spirituality experience might amplify the effect of mindfulness training in promoting flow. Accordingly, we propose the following hypothesis:

*H3*: Spirituality moderates the positive effect of mindfulness training on park visitors’ Flow.

Regarding the moderation of spirituality on “Flow → Life Hope”: Flow itself is a positive, immersive experience, but when intertwined with feelings of spirituality, its impact on building individual psychological resources may be more profound. Spirituality can imbue the flow process with a stronger sense of meaning and transcendence, making it not just an enjoyable activity but potentially an inspiring experience that broadens one’s perspective on life (Dulek and Stein, 2024). According to the Broaden-and-Build Theory, this spirituality-enhanced positive experience (flow) may more effectively build enduring psychological resources such as hope. Specifically, flow generated in an spirituality-inspiring context might more readily translate into positive expectations about future goals (pathways thinking) and the intrinsic motivation to achieve them (agency thinking). Accordingly, we propose the following hypothesis:

*H4*: Spirituality moderates the positive effect of park visitors’ Flow on their life hope.

#### 2.3.4 The multifaceted moderating role of sense of meaning in life

Sense of Meaning in Life (SMIL), as previously defined, is an individual’s subjective perception of their life’s purpose, value, and significance (Guo et al., 2025). It not only reflects the degree to which individuals possess a sense of meaning but may also influence how they benefit from positive experiences and interventions. In this experiment, we investigate how individuals’ baseline level of sense of meaning in life moderates the different pathways through which mindfulness training impacts life hope.

First, we re-examine the moderating role of sense of meaning in life on the direct path from “Mindfulness Training → Life Hope” (this was the original H4, now H4a): As previously argued, we anticipate that mindfulness training (Hossny et al., 2024) may have a stronger “compensatory” effect in enhancing life hope for individuals with a lower sense of meaning in life.

*H5*: Individuals’ baseline sense of meaning in life moderates the positive effect of mindfulness training (specifically the online modality, or the modality adopted in Experiment 4) on park visitors’ life hope.

Subsequently, our inquiry addresses how an individual’s sense of meaning in life (SMIL) may moderate the relationship between Mindfulness Training and Flow. It is plausible that the extent of perceived life meaning could affect an individual’s facility in achieving flow during mindfulness engagement (Hodes et al., 2023). One perspective suggests that those with greater SMIL may perceive mindfulness as a worthwhile endeavor consistent with their life objectives, potentially leading to more active participation and facilitating flow. Such individuals might more readily uncover deep experiences aligning with their values when mindfully interacting with natural environments (Rahimian, 2021). Conversely, for individuals reporting lower life meaning, possibly experiencing a lack of direction, the concentrated, present-moment awareness promoted by mindfulness—if it helps alleviate negative states—could represent a unique and deeply engaging state, thereby precipitating flow. Nevertheless, given that individuals with high SMIL often exhibit greater internal drive and purposefulness, they may more adeptly incorporate mindfulness practices (Ueberholz and Fiocco, 2022) within their established sense of meaning, potentially leading to more consistent flow experiences. Accordingly, we cautiously propose the following hypothesis:

*H6*: Individuals’ baseline sense of meaning in life moderates the positive effect of mindfulness training on park visitors’ Flow.

Finally, we investigate the moderating role of sense of meaning in life on the “Flow → Life Hope” path: When visitors experience flow in urban green spaces, whether this positive immersive experience effectively translates into an enhancement of life hope might also be influenced by their baseline level of sense of meaning in life (Martela et al., 2024). For individuals with a higher sense of meaning, Flows can be seen as further confirmation and enrichment of their meaningful lives, making it easier to internalize this positive state into optimistic future expectations and agency—that is, life hope. They can connect the enjoyment and control of flow with broader life goals. Conversely, for individuals with a lower sense of meaning, while flow itself is positive, the efficiency of its transformation into enduring life hope might be lower if a stable meaning framework is lacking to contain and interpret this experience (Swinney, 2023). Accordingly, we propose the following hypothesis:

*H7*: Individuals’ baseline sense of meaning in life moderates the positive effect of park visitors’ Flow on their life hope.

## 3 Overview of studies

To substantiate these propositions, four distinct experiments were undertaken in a sequential and logically building manner, moving from establishing a core effect to examining its mechanism and finally to delineating its boundary conditions. Initially, Experiment 1 was designed to determine the effect of mindfulness training (differentiating online and offline delivery) on life hope, thereby addressing hypotheses H1, H1a, H1b, and H1c. Subsequently, Experiment 2, also employing online versus offline mindfulness training paradigms, investigated the intermediary function of flow in the connection between mindfulness training and life hope (testing hypotheses H2, H2a, H2b, and H2c). The third experiment focused on how spirituality influences the mediating effect of flow within the mindfulness training-life hope linkage (addressing hypotheses H3 and H4). Finally, Experiment 4 aimed to explore the conditional effects of both sense of meaning in life and spirituality on flow’s mediation of the relationship between mindfulness training and life hope (testing hypotheses H5, H6, and H7). Demographic information is presented in Table 1. The video for the online mindfulness^1^. The research framework associated with the four Experiments is illustrated in Tables 1, 2.

**Table 1:** Demographic characteristics.

Variable	Item	Experiment 1 (*N* = 303)		Experiment 2 (*N* = 315)		Experiment 3 (*N* = 282)		Experiment 4 (*N* = 315)	
		Frequency	Proportion	Frequency	Proportion	Frequency	Proportion	Frequency	Proportion
Gender	Male	152	50.20 percent	158	50.20 percent	142	50.40 per cent	150	47.60 percent
	Female	151	49.80 percent	157	49.80 percent	140	49.60 percent	165	52.40 percent
Age	18–25 years old	88	29.00 percent	87	27.60 percent	83	29.40 percent	89	28.30 percent
	26–35 years old	138	45.50 percent	138	43.80 percent	131	46.50 percent	135	42.90 percent
	36–45 years old	26	8.60 percent	31	9.80 percent	19	6.70 percent	31	9.80 percent
	46–55 years old	26	8.60 percent	29	9.20 percent	32	11.30 percent	33	10.50 percent
	Over 56 years old	25	8.30 percent	30	9.50 percent	17	6.00 percent	27	8.60 percent
Education background	Primary school	26	14.20 percent	27	8.60 percent	24	8.50 percent	25	7.90 percent
	Junior high school	29	9.60 percent	27	8.60 percent	19	6.70 percent	26	8.30 percent
	Technical secondary school.	27	8.90 percent	25	7.90 percent	21	7.40 percent	34	10.80 percent
	College Specialty	26	8.60 percent	25	7.90 percent	25	8.90 percent	28	8.90 percent
	Undergraduate college	152	50.20 percent	160	50.80 percent	148	52.50 percent	156	49.50 percent
	Postgraduate	43	14.20 percent	51	16.20 percent	45	16.00 percent	46	14.60 percent

**Table 2:** Research framework.

Study	Study 1	Study 2	Study 3	Study 4
Purpose	Main effect of mindfulness training modality on life hope (H1a–H1c)	The mediating role of flow in the relationship between mindfulness training and life hope (H2a–H2c)	The moderating role of spirituality in the relationships among mindfulness training, flow, and life hope (H3–H4)	The multifaceted moderating role of sense of meaning in life (H5–H6)
Independent variable	Manipulated the online vs. offline of the mindfulness training	Manipulated the online vs. offline of the mindfulness training	Manipulated the online vs. offline of the mindfulness training	Manipulated the online vs. offline of the mindfulness training
Dependent variable	Life hope	Life hope	Life hope	Life hope
Mediators	__	Flow	Flow	Flow
Moderator	__	__	Spirituality	Spirituality sense of meaning in life
Methods	ANOVA	ANOVA PROCESS Model 4	ANOVA PROCESS Model 8	ANOVA PROCESS Model 63
Results	Supported (H1a–H1c)	Supported (H2a–H2c)	Supported (H3–H4)	Supported (H5–H6)

## 4 Experiment 1: testing the main effect

The primary aim of Experiment 1 was to preliminarily examine our focal hypothesis regarding how the format of mindfulness training (Online vs. offline) influences the enhancement of life hope among urban park visitors.

### 4.1 Method

Participants were recruited through two distinct channels. Participants for the Online Mindfulness group (*n* = 149) were recruited via the Credamo platform (http://www.credamo.c, a professional domestic online survey platform). Participants for the offline Park Mindfulness group (*n* = 154) were recruited from a public park located in Shenzhen City, Anhui Province, China. To further ensure the validity of the data, the recruitment process for both groups was completed across three distinct phases. Two participants who failed an attention check were excluded from the analysis, resulting in a final sample size of 303. Detailed demographic information for the sample is presented in Table 1.

The experimental procedures strictly adhered to ethical guidelines, commencing only after informed consent was obtained from all participants, who initially completed a battery of baseline questionnaires assessing their hope and relevant psychological variables. Subsequently, participants were randomly assigned to either an Online Mindfulness group or an offline Park Mindfulness group. Those in the OM group were individually situated in a park setting to watch a standardized online mindfulness training video (approximately 15 min) guiding them through exercises such as body scans and mindful breathing. Conversely, IPM group participants were directed to a pre-selected quiet area within an urban park where a trained volunteer facilitated a group mindfulness session of equivalent duration, with core instructions mirroring the OM video while also encouraging awareness of surrounding natural elements. To ensure comparability of intervention content, core mindfulness principles and practice duration remained consistent across conditions, the primary distinction being the mode of delivery (online vs. offline). Finally, all participants were fully debriefed on the study’s purpose and received appropriate compensation (3 RMB). After completing the reading, participants are required to answer a measurement question about life hope: “When I am in a predicament, I will come up with various ways to get myself out of it.” (1 = strongly disagree, 7 = strongly agree) (Snyder, 2002; Snyder et al., 1991). The manipulation of the independent variable was based on the research findings of other scholars (Bossi et al., 2022; Pickett et al., 2024).

### 4.2 Results

To compare life hope levels based on the mindfulness training format, an Analysis of Variance (ANOVA) was employed. Findings revealed that individuals in the Online Mindfulness condition (*M* = 5.63, SD = 1.20) demonstrated significantly greater life hope than those in the offline Park Mindfulness condition (*M* = 5.27, SD = 1.03), *F*(1, 301) = 7.85, *p* < 0.05. These results lend support to Hypothesis 1 H1 and validated hypotheses H1a–H1c.

### 4.3 Discussion

While Experiment 1 successfully established the superior efficacy of online mindfulness in boosting life hope (H1), it left a key question unanswered: what psychological pathway drives this effect? To move from observing what works to understanding how it works, we posited that the online format might be more conducive to inducing a state of flow. Accordingly, Experiment 2 was designed to test this proposed mediation, thereby delving deeper into the mechanism while re-examining the robustness of the main effect.

## 5 Experiment 2: testing for mediation

The primary aim of Experiment 2 was to thoroughly investigate the mediating role of flow in the relationship between mindfulness training formats (Online vs. Offline) and the enhancement of life hope among urban park visitors. This experiment sought to explore whether the state of flow is a key factor that explains how different formats of mindfulness training affect individuals’ sense of hope.

### 5.1 Method

Participants were recruited through two distinct channels. Participants for the Online Mindfulness group (*n* = 161) were recruited via the Credamo platform (http://www.credamo.c, a professional domestic online survey platform). Participants for the offline Park Mindfulness group (*n* = 154) were recruited from a public park located in Shenzhen City, Anhui Province, China. To further ensure the validity of the data, the recruitment process for both groups was completed across four distinct phases. This recruitment strategy yielded an initial sample of 320 participants. Five participants who failed an attention check were excluded from the analysis. The remaining participants were then randomly assigned to either the Online Mindfulness group (*n* = 161) or the offline Park Mindfulness group (*n* = 154). Detailed demographic information for the sample is presented in Table 1. The experimental procedure was identical to that of Experiment 1. After completing the reading, participants are required to answer measurement questions about life hope, such as “I feel energized and striving to achieve my goals.” (1 = strongly disagree, 7 = strongly agree) (Snyder, 2002; Snyder et al., 1991) and flow measurement questions, such as “I am happy watching WTT.” (1 = strongly disagree, 7 = strongly agree) (Pai et al., 2025). The manipulation of the independent variable was based on the research findings of other scholars (Bossi et al., 2022; Pickett et al., 2024).

### 5.2 Results

Main Effect Test: Consistent with Hypothesis 1 (H1), an ANOVA revealed a significant main effect of mindfulness training format on life hope. The Online Mindfulness group (*M* = 6.00, SD = 4.79) reported significantly higher life hope than the offline Park Mindfulness group (*M* = 4.79, SD = 0.57), *F*(1, 313) = 176.26, *p* < 0.001.

**Figure 1:**
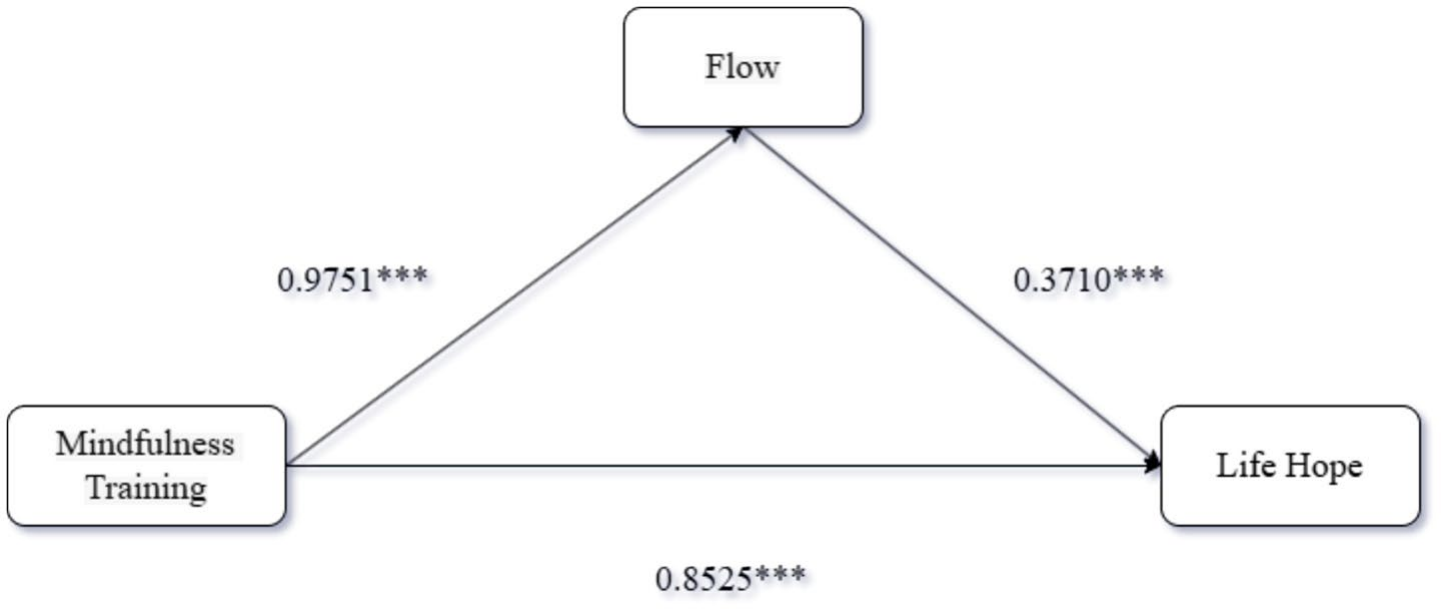
Results of Experiment 2.

Mediation Analysis: Using PROCESS Model 4 (Hayes, 2018) with 5,000 bootstrap samples, we examined flow as a mediator between training format and life hope. The analysis indicated that training format significantly predicted both flow (*β* = 0.98, 95% CI [0.74, 1.21]) and life hope (*β* = 0.85, 95% CI [0.68, 1.03]). Flow also significantly predicted life hope (*β* = 0.37, 95% CI [0.30, 0.44]). The indirect effect of training format on life hope via flow was significant (indirect effect = 0.36, 95% CI [0.25, 0.49]), as the confidence interval excluded zero, supporting flow’s mediating role. Validated hypotheses H2a to H2c. The results are presented in Figure 1.

### 5.3 Discussion

Experiment 2 successfully illuminated how the effect operates by establishing flow as a key mediator. This finding, however, leads to a more nuanced question: For whom is this mechanism most effective, and under what conditions? Individual differences might moderate the strength of this mediated pathway. We theorized that spirituality, given its conceptual link to both mindfulness and transcendent experiences like flow, could be a critical moderator. Therefore, Experiment 3 was designed to test the moderating role of spirituality in this established mediation model.

## 6 Experiment 3: testing for moderated mediation

Experiment 3 aimed to test our focal hypothesis regarding the impact of mindfulness training (Online vs. offline) on life hope, re-examining the main effect. We also investigated whether spirituality moderated the effect of mindfulness training on life hope.

### 6.1 Method

Participants were recruited through two distinct channels. Participants for the Online Mindfulness group (*n* = 142) were recruited via the Credamo platform (http://www.credamo.c, a professional domestic online survey platform). Participants for the offline Park Mindfulness group (*n* = 140) were recruited from a public park located in Shenzhen City, Anhui Province, China. To further ensure the validity of the data, the recruitment process for both groups was completed across two distinct phases. This recruitment strategy yielded an initial sample of 290 participants. Eight participants who failed an attention check were excluded from the analysis. The remaining participants were then randomly assigned to either the Online Mindfulness group (*n* = 142) or the offline Park Mindfulness group (n = 140). Detailed demographic information for the sample is presented in Table 1. The experimental procedure was identical to that of Experiment 1. After completing the reading, participants are required to answer questions about the measurement of life hope, such as “There are many solutions to any problem I encounter now.” (1 = strongly disagree, 7 = strongly agree) (Snyder, 2002; Snyder et al., 1991) and the flow measurement question, “I think time passes very quickly when watching WTT.” (1 = strongly disagree, 7 = strongly agree) (Pai et al., 2025) Spiritual measurement question “During the visit, I felt peaceful.” (1 = strongly disagree, 7 = strongly agree) (Tsaur et al., 2024). The manipulation of the independent variable was based on the research findings of other scholars (Bossi et al., 2022; Pickett et al., 2024).

### 6.2 Results

Main Effect Test: Consistent with Hypothesis 1 (H1), an ANOVA revealed that the Online Mindfulness group (*M* = 6.20, SD = 0.64) reported significantly higher life hope than the offline Park Mindfulness group (*M* = 5.97, SD = 1.07), *F*(1, 280) = 4.76, *p* = 0.030.

**Figure 2:**
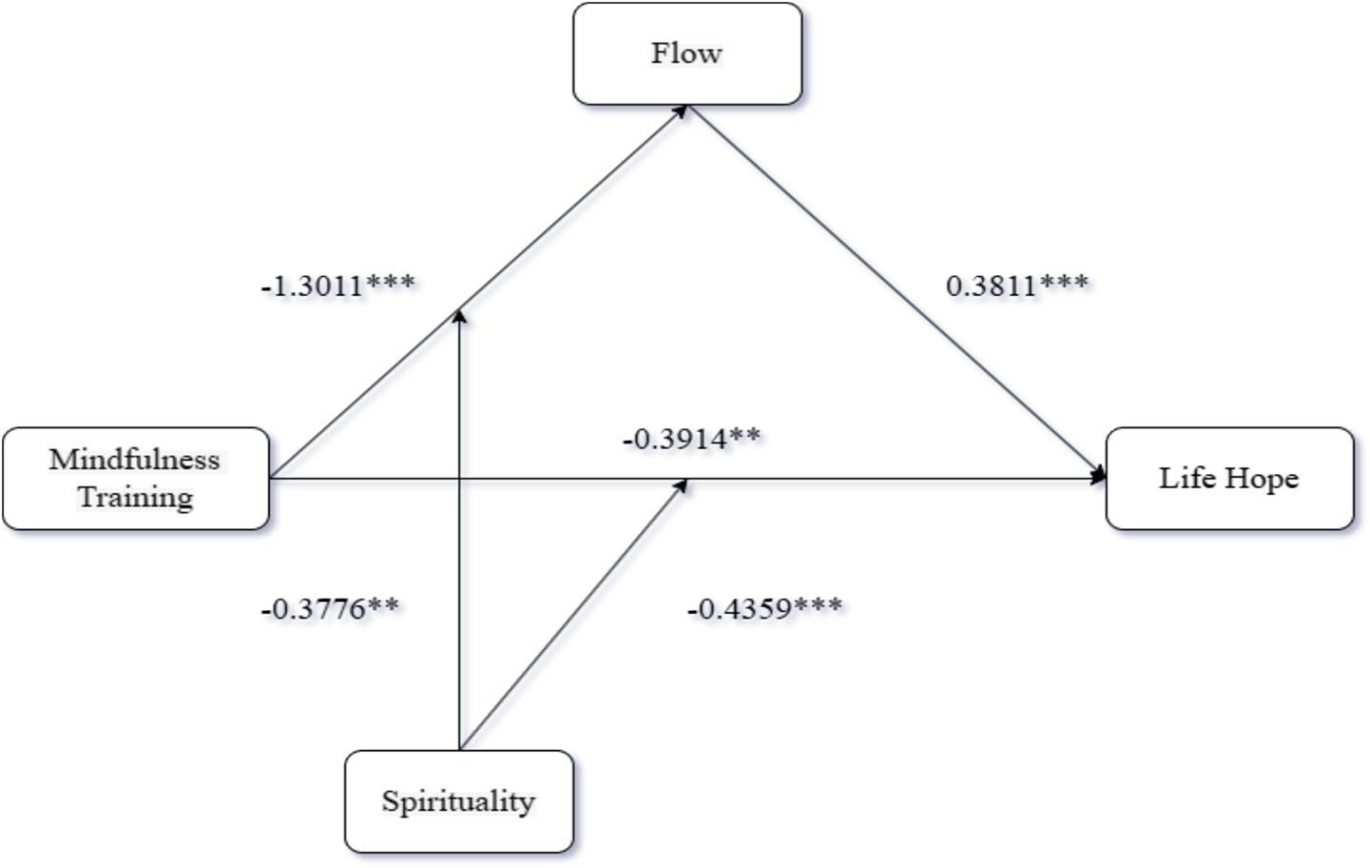
Results of Experiment 3.

**Figure 3:**
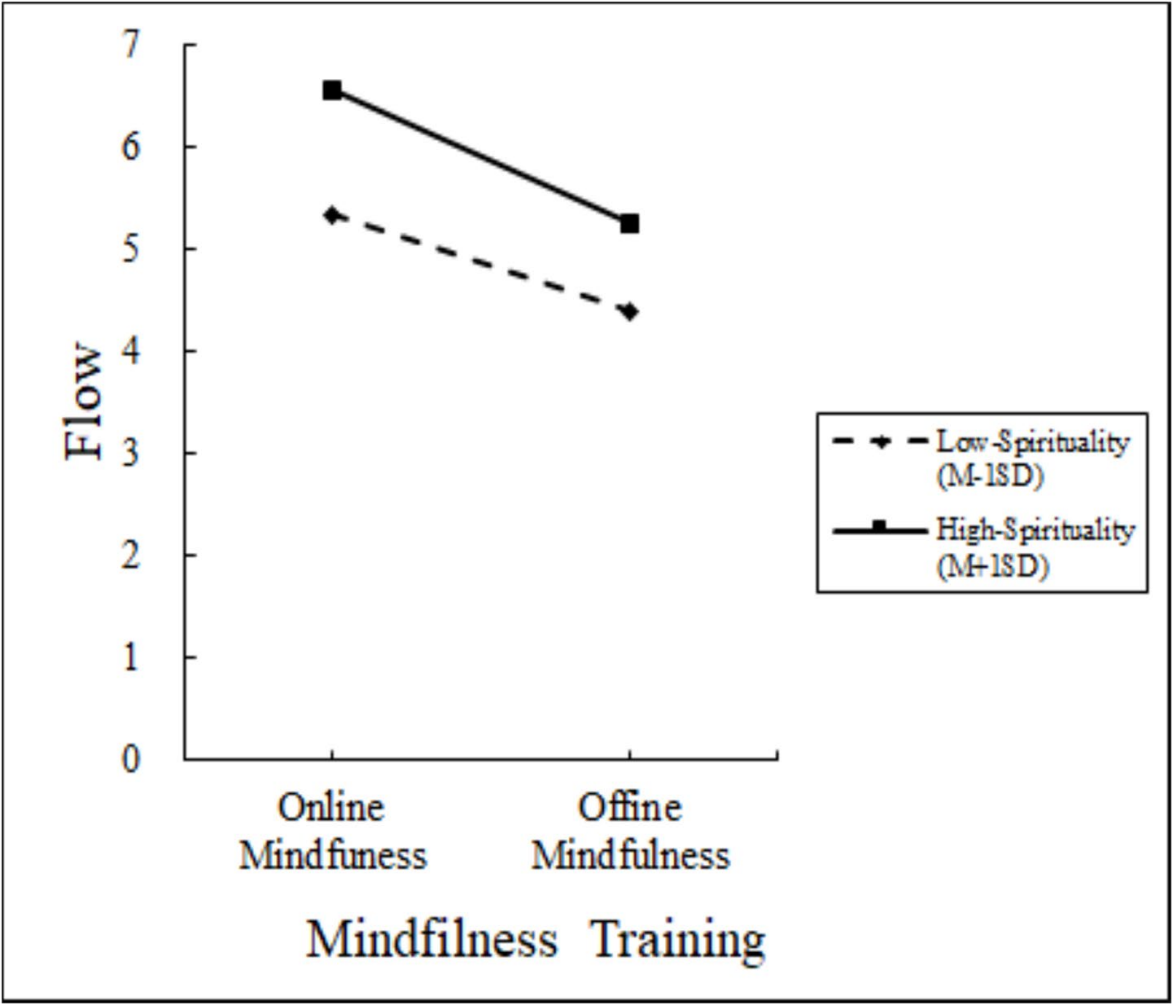
Moderator of Experiment 3.

**Figure 4:**
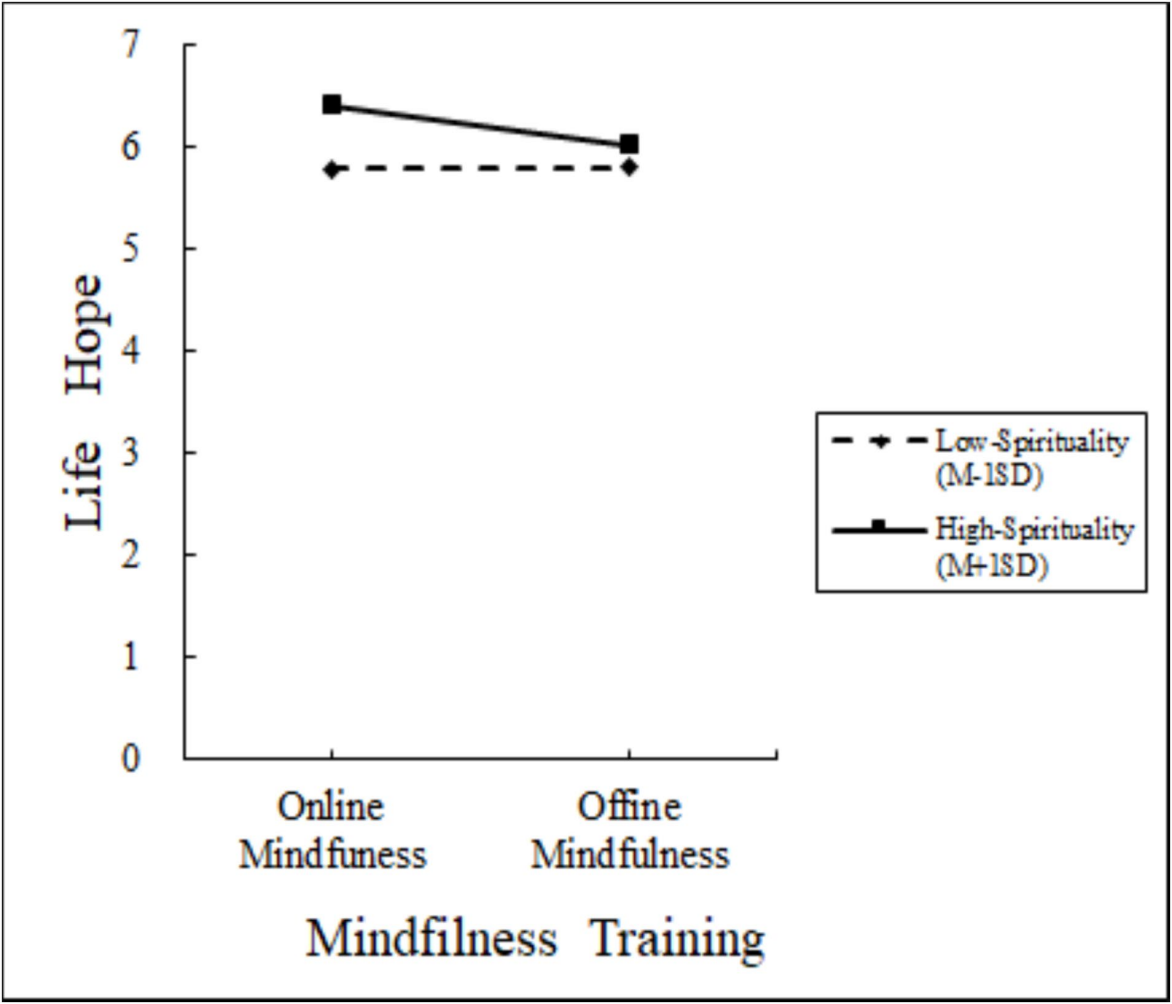
Moderator of Experiment 3.

Moderated Mediation Analysis: Using PROCESS Model 8 (Hayes, 2018; 5,000 bootstraps), we tested spirituality as a moderator of the mediating role of flow between mindfulness training format and life hope. Mindfulness training significantly predicted flow (*β* = −1.30, 95% CI [−1.58, −1.02]) and had a significant direct effect on life hope (*β* = −0.39, 95% CI [−0.63, −0.16]). Flow also significantly predicted life hope (*β* = 0.38, 95% CI [0.29, 0.47]). Crucially, spirituality significantly moderated the mindfulness training-flow pathway (interaction: *β* = −0.38, 95% CI [−0.69, −0.07]). The index of moderated mediation (Index = −0.44, 95% CI [−0.67, −0.20]) confirmed that spirituality moderated the indirect effect of training on life hope via flow. Spirituality also significantly moderated the direct relationship between mindfulness training and life hope (interaction: *β* = −0.14, 95% CI [−0.33, −0.02]). Validated hypotheses H3 to H4. The results are presented in Figures 2–4.

### 6.3 Discussion

Experiment 3 successfully demonstrated that spirituality moderates the mediated pathway, providing crucial insight into the conditional nature of our model. While this finding is significant, a robust theoretical framework requires that its principles hold across more than a single psychological construct. To further test the robustness and generalizability of our model, it is important to examine whether other, conceptually distinct yet relevant, individual differences also shape this effect. We identified meaning in life as another key potential moderator. Therefore, Experiment 4 was designed to test the moderating role of meaning in life, aiming to replicate the moderated mediation model with a new variable.

## 7 Experiment 4: testing for moderated mediation

Experiment 4 aimed to test our focal hypothesis regarding the impact of mindfulness training (Online vs. offline) on life hope, re-examining the main effect. We also investigated whether meaning in life and spirituality moderated the effect of mindfulness training on life hope.

### 7.1 Method

Participant recruitment yielded 310 individuals through the Credamo platform (http://www.credamo.c, a specialized domestic online survey provider). Three participants were subsequently excluded from the analysis due to failing an attention check. The remaining participants were then subjected to random assignment, allocating them to either the Online Mindfulness condition (n = 154) or the offline Park Mindfulness condition (n = 153). A comprehensive overview of the sample’s demographic characteristics is available in Table 1. The experimental procedure was identical to that of Experiment 1. Finally, participants were fully debriefed regarding the study’s purpose and received appropriate compensation (CNY 2). After completing the reading, participants are required to answer a measurement question about their hope for life: “At this moment, I believe I am quite successful.” (1 = strongly disagree, 7 = strongly agree) (Snyder, 2002; Snyder et al., 1991) and a flow measurement question: “I feel that when I watch WTT, my attention is entirely focused on it.”(1 = strongly disagree, 7 = strongly agree) (Pai et al., 2025) and the measurement question of spirituality “During the visit, I felt a sense of comfort in my soul.” (1 = strongly disagree, 7 = strongly agree) (Tsaur et al., 2024) and the measurement question of meaning in life “I understand my own meaning in life very well.” (1 = strongly disagree, 7 = strongly agree) (Steger et al., 2006). The manipulation of the independent variable was based on the research findings of other scholars (Bossi et al., 2022; Pickett et al., 2024).

### 7.2 Results

Main Effect Test: An ANOVA, with mindfulness training format as the independent variable and life hope as the outcome, was performed. This analysis revealed that the Online Mindfulness group exhibited significantly greater life hope (*M* = 6.06, SD = 0.97) compared to the offline Park Mindfulness group (*M* = 5.71, SD = 1.43), *F*(1, 305) = 6.19, *p* < 0.05. This result was in support of Hypothesis 1 (H1).

**Figure 5:**
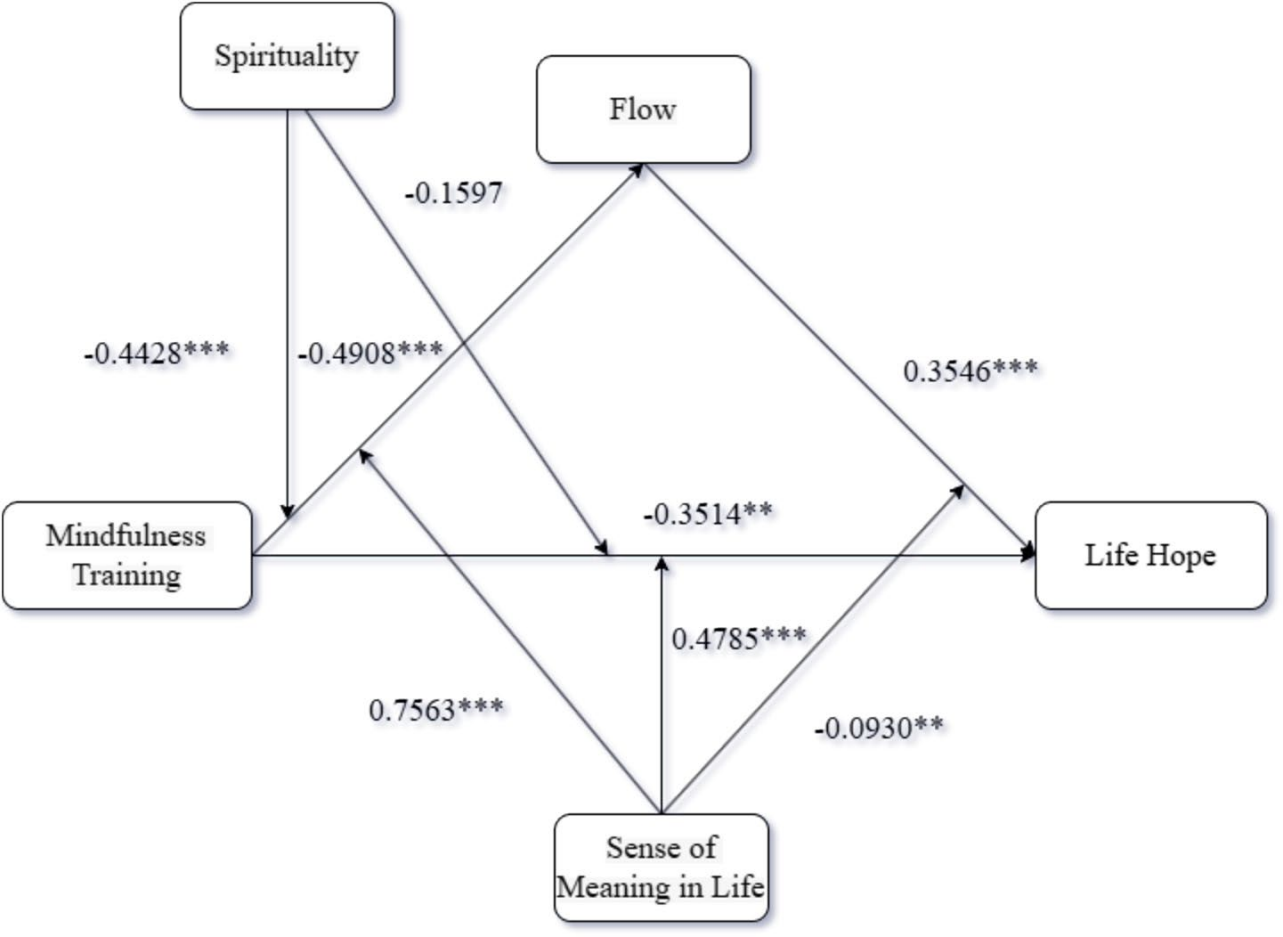
Results of Experiment 4.

Moderated Mediation Analysis. To investigate moderated mediation, PROCESS Model 63 (Hayes, 2018) with 5,000 bootstrap samples was employed. Mindfulness training format served as the independent variable, flow as the mediator, meaning in life and spirituality as moderators, and life hope as the dependent variable. Findings indicated a significant effect of mindfulness training on flow (*β* = −0.49, 95% CI [−0.70, −0.28]) and a significant direct effect on life hope (β = −0.35, 95% CI [−0.58, −0.12]). Flow also significantly predicted life hope (*β* = 0.35, 95% CI [0.25, 0.46]). Regarding moderation by meaning in life, its interaction with mindfulness training was a significant predictor of flow (interaction term: *β* = 0.76, 95% CI [0.50, 1.01]). Meaning in life also demonstrated a significant moderating effect on the direct pathway from mindfulness training to life hope (interaction term: *β* = 0.48, 95% CI [0.23, 0.73]). Validated hypotheses H5 to H7. Furthermore, the interaction between flow and meaning in life significantly predicted life hope (interaction term: *β* = −0.09, 95% CI [−0.16, −0.02]). Concerning spirituality’s moderating role, its interaction with mindfulness training significantly predicted flow (interaction term: β = −0.44, 95% CI [−0.69, −0.19]). The interaction between mindfulness training and spirituality, however, did not significantly predict life hope (interaction term: *β* = −0.16, 95% CI [−0.40, 0.08]). Validated hypotheses H5 to H7. The results are presented in Figure 5.

### 7.3 Discussion

Experiment 4 replicated the main effect, indicating that online mindfulness led to higher life hope than offline park mindfulness, despite the numerical means suggesting the opposite direction. The moderated mediation analysis confirmed flow as a mediator. Meaning in life significantly moderated several paths: the impact of mindfulness training on flow, its direct effect on life hope, and the flow-life hope relationship. Spirituality also moderated the mindfulness training-flow link. However, distinct from meaning in life, spirituality did not significantly moderate the direct mindfulness training-life hope pathway. These findings highlight the nuanced roles of meaning in life and spirituality in the effectiveness of different mindfulness training formats.

## 8 General discussion

### 8.1 Experimental summary

A four-experiment research program investigated how mindfulness training influences park visitors’ life hope (Barnhofer et al., 2021), examining direct effects, the mediating role of flow, and moderating influences of spirituality and sense of meaning in life. The overarching aim was to understand how different training modalities affect life hope and to clarify the underlying psychological processes and contextual factors (Liu, 2024). Initially, Experiment 1 (H1, H1a-c) established that the training mode (online vs. offline) significantly impacts life hope and validated both approaches. Subsequently, Experiment 2 (H2, H2a-c) demonstrated that flow significantly mediates the relationship between engaging in mindfulness training and the subsequent enhancement of life hope. Experiment 3 then investigated spirituality’s moderating influence on this mediation (H3, H4), revealing that visitor spirituality altered flow’s mediating effect, though Hypothesis H4 was not empirically supported. Finally, Experiment 4 (H5-H7) further elucidated how individual differences in sense of meaning in life (Chereches et al., 2025), in conjunction with or parallel to spirituality, moderate flow’s mediating role in the mindfulness-life hope relationship, thus providing a more nuanced understanding of the intervention’s boundary conditions. Collectively, these studies offer a comprehensive account of how online and offline mindfulness training can foster life hope, highlighting flow as a key mediator and identifying spirituality and sense of meaning in life as crucial moderators of this process.

Beyond these findings, our research offers several significant theoretical contributions. First, our work integrates and empirically validates a comprehensive moderated mediation model, connecting mindfulness practice to life hope. By demonstrating that flow acts as a key mediating mechanism, we elucidate how mindfulness translates into enhanced hope, thereby refining both mindfulness and flow theories by establishing a direct causal link between a contemplative practice and the experience of optimal engagement. Second, we significantly extend the understanding of the boundary conditions under which mindfulness interventions are effective. By identifying spirituality and sense of meaning in life as key moderators, our findings advance the “person-environment fit” perspective in positive psychology, moving beyond a “one-size-fits-all” approach to show for whom and under what psychological conditions the intervention is most potent. Finally, our comparative analysis of online versus offline modalities challenges the implicit assumption that place-based interventions are inherently superior, suggesting that the core mechanisms of psychological theories (e.g., inducing flow) can be effectively activated across different delivery platforms, thus broadening their applicability in a digital world.

### 8.2 Theoretical contribution

The findings of this multi-experiment research program offer several significant theoretical contributions to the intersecting fields of environmental psychology, positive psychology, and the experiment of digital well-being interventions. Firstly, this research advances our understanding of how distinct psychological theories can be synergistically integrated to explain the enhancement of complex positive outcomes like life hope within specific socio-ecological contexts (Magliocca et al., 2023). While Attention Restoration Theory elucidates why urban green spaces (UGS) are restorative, and the Broaden-and-Build Theory explains how positive experiences build resources, and Hope Theory defines what specific resource (life hope) is cultivated (Stahl and Sokolov, 2024), few studies have empirically woven these frameworks together to examine an active intervention like online mindfulness (Csonka et al., 2022). Our findings extend the work of Kaplan and Kaplan, Fredrickson, and Snyder by demonstrating a more comprehensive model where the restorative setting (ART) facilitates a state conducive to mindfulness, which then, through mechanisms like flow (Li et al., 2024), fosters the cognitive components of hope. This integrated perspective contributes to a more nuanced theoretical foundation within environmental psychology (by showing how UGS can be actively leveraged for targeted psychological gains beyond passive restoration) and positive psychology (by detailing a contextualized pathway to hope cultivation through accessible digital means) (Driver, 2024).

Secondly, this experiment significantly contributes to the burgeoning field of mindfulness research, particularly concerning digitally-delivered interventions in natural settings, by identifying key psychological mechanisms and boundary conditions. While the general benefits of mindfulness are well-documented (Dong et al., 2023), our research moves beyond simply affirming its efficacy. By establishing Flow as a significant mediator (Experiment 2), we provide empirical support for how mindfulness, especially when potentially enhanced by the UGS context, translates into increased life hope (Kou et al., 2023). This deepens the theoretical understanding of mindfulness by highlighting an experiential pathway. Furthermore, by demonstrating the moderating roles of spirituality and sense of meaning in life (Cappannini et al., 2024), our findings delineate crucial contextual and individual difference factors that shape the effectiveness of the intervention. This contributes novel insights to mindfulness science and positive psychology intervention research by specifying for whom (e.g., individuals with varying levels of SMIL) and under what experiential conditions (e.g., the presence of spirituality) online mindfulness in UGS is most likely to foster life hope, thus informing more personalized and effective intervention design (Tobiano et al., 2024).

Thirdly, this research makes a specific contribution to Hope Theory (Clemente, 2025) by empirically demonstrating a novel, accessible, and context-sensitive intervention strategy for proactively cultivating life hope among the general population (park visitors). Much of the research on hope has focused on its correlates, its role in coping with adversity, or interventions in clinical settings (Zanini et al., 2024). Our experiment extends this body of work by showing that online mindfulness, particularly when situated within the restorative and potentially spirituality-inspiring context of urban green spaces, can serve as a practical tool for enhancing life hope as a positive psychological resource in a non-clinical, everyday setting (Bagdiūnien et al., 2025). This finding is significant for positive psychology by expanding the repertoire of evidence-based hope-enhancing strategies, and it also contributes to the fields of tourism/leisure studies and public health psychology by underscoring how readily accessible natural environments, augmented by digital tools (Datta et al., 2024), can be transformed into platforms for substantive psychological well-being improvements, moving beyond mere recreation to targeted positive psychological development (Wang et al., 2025).

### 8.3 Practical implications

The findings of this research program not only deepen our theoretical understanding but also offer several actionable insights. Specifically, our primary finding that digitally-guided online mindfulness surpassed self-guided offline practice in enhancing life hope (Experiment 1) provides a counter-intuitive but powerful directive for urban planners and park managers. Rather than viewing technology as a distraction, it should be strategically integrated into Urban Green Spaces (UGS) to create “therapeutic landscapes.” A concrete application would be embedding QR codes in tranquil park locations, linking visitors to curated mindfulness exercises tailored to their environment. Furthermore, our discovery that ‘flow’ is a critical mediator (Experiment 2) informs the content of these digital tools; they must be designed to foster absorption by balancing challenge, providing clear goals, and offering feedback. Finally, our moderation findings (Experiments 3 & 4) reveal that these benefits are amplified for individuals with higher spirituality and meaning in life, highlighting the need for personalization (). For digital health developers, this translates into creating adaptive apps. For instance, an app could use a brief onboarding questionnaire to assess a user’s sense of meaning, then recommend either advanced contemplative exercises for high-scorers or foundational, meaning-making modules for those scoring lower (). By directly linking these specific findings to actionable strategies, we can more effectively translate research into tangible improvements in public mental health ().

Secondly, for developers of digital health products and mental health practitioners, our findings provide a precise, evidence-based roadmap for creating more effective interventions. Our discovery that ‘flow’ acts as the crucial psychological mechanism enhancing life hope (Experiment 2) translates directly into a core design principle. Rather than offering generic relaxation scripts, the primary objective should be to engineer experiences that foster deep absorption. Therefore, digital mindfulness scripts should intentionally incorporate elements that guide users to minutely observe natural details, feel a profound connection with nature, or experience its grandeur (), as our model suggests these activities are effective precisely because they induce a state of flow.

Furthermore, our moderation findings—that the intervention’s benefits are significantly amplified for individuals with higher spirituality and meaning in life (Experiments 3 and 4)—point directly to the necessity of personalization. This insight provides a strong rationale for creating context-and person-aware applications. For example, an app could leverage GPS to identify when a user is in a park and then, informed by our results, recommend a tailored practice based on their psychological profile. A user with high spirituality might receive a guide on awe and interconnectedness, while another might receive a foundational sensory awareness exercise. This transforms a generic app into a potent adjunctive tool for mental health professionals (), allowing them to prescribe digital interventions that are not only synergistic with nature exposure but are also personalized to maximize therapeutic impact.

Thirdly, for public health agencies and policymakers, our findings offer a compelling, evidence-based case for a highly cost-effective and scalable public health strategy. Our central finding that a digitally-delivered mindfulness intervention was not just an alternative, but was more effective than a self-guided park visit, is of profound policy relevance. It demonstrates that significant mental health benefits can be delivered at scale through digital platforms, which have minimal marginal costs. This proven effectiveness and scalability provide a strong rationale for public health initiatives. For instance, authorities could launch campaigns like an “Urban Oasis, Mindful Moment,” confidently promoting the use of local parks combined with officially endorsed online mindfulness resources. Crucially, the accessibility of this digital model means it can reach diverse populations, including those with mobility or time constraints. This makes the “UGS + online mindfulness” model ideal for integration into broad health frameworks, such as student mental wellness programs or corporate employee assistance programs (Angel et al., 2025), offering a seamless way to embed mental health support into daily life and contribute to building more resilient cities (Encuentra et al., 2024).

### 8.4 Limitations and future directions

While this experiment offers valuable contributions, several limitations warrant attention and guide future research. Firstly, the primary reliance on self-report measures introduces potential subjective biases, and the cross-sectional nature of some designs limits causal inference strength. Future research should integrate objective data, such as physiological indicators and behavioral observations, and employ longitudinal designs to more accurately assess intervention effects and their durability (Saito et al., 2024). Secondly, the focus on specific types of urban green spaces and online mindfulness restricts generalizability. Future studies should systematically compare the effects within varied UGS contexts and explore other digital interventions (e.g., nature-based positive psychology apps) in similar settings to broaden theoretical and practical frameworks. Lastly, the long-term effects and optimal “dosage” of the intervention were under explored (Smith et al., 2024). Future work urgently needs to conduct longer-term follow-ups and design parametric studies to determine the optimal practice regimens for sustaining and enhancing life hope, thereby providing empirical support for personalized interventions and exploring mechanisms for sustained benefits. These endeavors will deepen our understanding of the complex “green space-digital intervention-positive psychology” nexus and drive the development of more effective well-being promotion strategies (Cho and Frizzell, 2024). Additionally, while the current findings may be affected by baseline differences from different recruitment channels (online and offline), future research should recruit participants within the same city to reduce baseline interference.

## Data Availability

The original contributions presented in the study are included in the article/supplementary material, further inquiries can be directed to the corresponding author.

## Footnotes

^1^ https://www.youtube.com/watch?v=8v45WSuAeYI
