# **Correction:** Fostering life hope in urban green spaces through brief online mindfulness: findings from four studies with park visitors

**DOI:** 10.3389/fpsyg.2025.1676202

**Published:** 2025-08-13

**Authors:** Mengke Luo, Weiyi Zhang

**Affiliations:** ^1^Macau University of Science and Technology, Avenida WaiLong, Taipa, Macao SAR, China; ^2^Huainan Normal University, West Dongshan Road, Tianjiaan District, Huainan, China

**Keywords:** urban green spaces, mindfulness training, life hope, flow, sense of meaning in life

Author Weiyi Zhang was erroneously assigned to affiliation 1 “Macau University of Science and Technology, Avenida WaiLong, Taipa, Macao SAR, China.” This affiliation has now been removed for author Weiyi Zhang.

The original version of this article has been updated.

